# Should "Sisters" and "Nurses" Marry?

**Published:** 1887-01-01

**Authors:** 


### Should "Sisters" and "Nurses" Marry?

### THE STORY OF "SISTER OLIVE."

As I am a Matron of what is at present a small hospital, I do not expect my opinion to carry much weight, but, far from being ashamed to see The Hospital on my table, I look forward to its weekly arrival with pleasure. My nurses have their own copy, and I have not found that the "moral tone" of this particular institution is at all being lowered by the interesting story, "Sister Olive."

Sister Edith.

Princess Alice Memorial Hospital, Eastbourne, Christmas, 1886.

Will you allow me to say a few words with regard to the subject of Miss Wood's letter? When the tale, or romance, first appeared, I expressed an earnest hope that some one, in the interests of The Hospital publication, would write a protest against what appears to me, to say the least, a most injudicious and undesirable article. Without entering upon any details or particular circumstances therein narrated, or going into the question of morals, it surely cannot be well to put in the fore-front of nursing-work such thoughts, and possibly motives, as are here suggested. Those who look unkindly on the present fashion, as they call it, are only too ready to ascribe unworthy motives for young ladies undertaking this solemn profession and work; and what can give a better handle to such criticism than tales like the present? So cordially do I agree with all Miss Wood has said, that I have had a strong objection to circulating the present numbers amongst our young nurses, whose thoughts must be turned from serious devotion to their work, by so exciting, and, may we hope, unnatural a narrative. I feel sure that hospital managers will agree with us that their difficulties are at present great enough without being enhanced by a style of literature likely to promote, instead of checking them, and which is brought forward in a publication especially designed for circulation amongst hospitals, and which is largely read by nurses.

Louisa Twining.

Dec. 24th.

Miss Twining's letter raises two questions of great importance, to answer which will be to answer many other related questions. The first is this: Do ladies and others who enter upon the nursing profession give up their right or their wish to marry, or to be approached with a view to marriage? The second is wider, and affects an infinitely larger number of persons. It is this: Is the art of the novelist and the story-teller moral and good? Miss Twining would, we suppose, approach both these questions from the standpoint of a sincere belief in the Christian faith. That also is our own standpoint. In the first place: Do ladies and others who become nurses give up their right or their wish to marry? It is an important question, as old as conventual institutions and conventual discipline, and not to be answered in a rash or inconsiderate spirit. We think, however, that most ladies and others who take up the profession of nursing as a calling, enter upon it with the same objects as they enter upon other and more obviously secular callings—viz., for the purpose of earning a livelihood at a congenial occupation. If this view be correct, then they probably no more think of debarring themselves from marriage by becoming nurses than they do by becoming telegraph clerks, or secretaries, or lady doctors. The idea of separating themselves entirely to that work by vow, either expressed or implied, probably does not enter into consideration except in the case of a limited number of the more ascetically-minded among nurses. If ladies prefer to separate themselves absolutely for life, either by vow or otherwise, there can be no objection; but we think the majority of lady nurses neither intend nor desire to do so. As we have said, they enter upon the nursing profession because it offers them a congenial occupation; and, provided they honourably fulfil the engagements into which they may enter with hospitals and other training institutions, they feel themselves at full liberty to revert to the ordinary life of educated women, either by marriage or otherwise. But, if they feel themselves at liberty to marry, then it follows, as a corollary, that they feel themselves at liberty to be approached with a view to marriage; and it equally follows that, if they may be approached by men generally, so also they may be approached by doctors, and by house-physicians and surgeons. We hold —and our experience is certainly not limited—that conduct and the sense of propriety in resident medical men, and in lady and other nurses, is at least on a level with that of the same class of persons elsewhere; and, therefore, that they are as worthy of trust and confidence as the same class elsewhere; and therefore, also, that the same rules of propriety—the same and no other—must govern them as are applied to similar classes elsewhere. Hospitals are not convents, but public institutions where men and women have to meet, and to associate together openly in the performance of certain duties. If it sometimes happens, as it undoubtedly does, that men and women so meeting esteem each other, and that their esteem is followed by marriage, where is the injury to morals or the offence against propriety? We cannot help thinking that if Miss Twining and others—whose judgment we esteem most highly—would look a little deeper into the subject, they would come to the same conclusion as we have done—viz., that in hospitals, as elsewhere, good and honourable men and women will continue to be good and honourable whether discipline be strict or moderate; and, that men and women who are not good and honourable will not be made so by hospital discipline; and, indeed, are out of place altogether in hospitals. With regard to the second question: whether or not the art of the novelist and story-teller is favourable to good morals, we have left ourselves no space for an adequate discussion. We may simply say, that the art is as old and as wide as literature itself; that it is common to sacred and profane literature alike; and that some of the most beautiful and touching examples of the story-teller's art which the world has ever seen are to be found in the New Testament, in the parables of our Lord.—[Ed. T. H.]

